# Supplementary material for: Resolving the full spectrum of human genome variation using Linked-Reads
Source: Genome Res. 2019 Apr;29(4):635–45. doi: 10.1101/gr.234443.118 (PMC6442396; doi:10.1101/gr.234443.118)
Supplement: Supplemental Material [file supp_gr.234443.118_Supplemental_longranger-2.2.zip › longranger-2.2.tar.bz2!/lr2.2-public/lib/go/src/code.google.com/vitess/docs/resources/coreos-meetup-2016-01-27.pdf]

YouTube Vitess

Anatomy of a Distributed Database on Kubernetes

CoreOS Meetup

January 27, 2016

Anthony Yeh, Software Engineer, YouTube

Outline

1. Vitess
2. Kubernetes
3. Databases on Kubernetes
4. Vitess on Kubernetes

Vitess

Vitess 1.0

golang.org

- YouTube is a MySQL app
- Vitess started in YouTube data centers
  - Connection-pooling proxy
- Early adopter of Go (golang.org)
  - First commit (in original repo) in 2010
  - 2 years before Go 1.0
  - Cheap connections, goroutines
- In production at YouTube since 2011

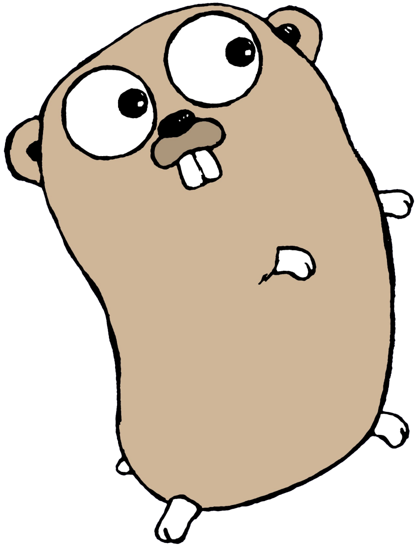

Renee French (not pictured)

Vitess 1.5

- YouTube moved into Borg[1]
  - Google MySQL
- Adapted Vitess to Borg environment
  - Dynamically scheduled container cluster
- Over time, Vitess evolved within Borg
  - Database protection
  - Query rewriting/blacklisting
  - Row-based cache
  - Shard routing
  - Cluster management
  - Monitoring

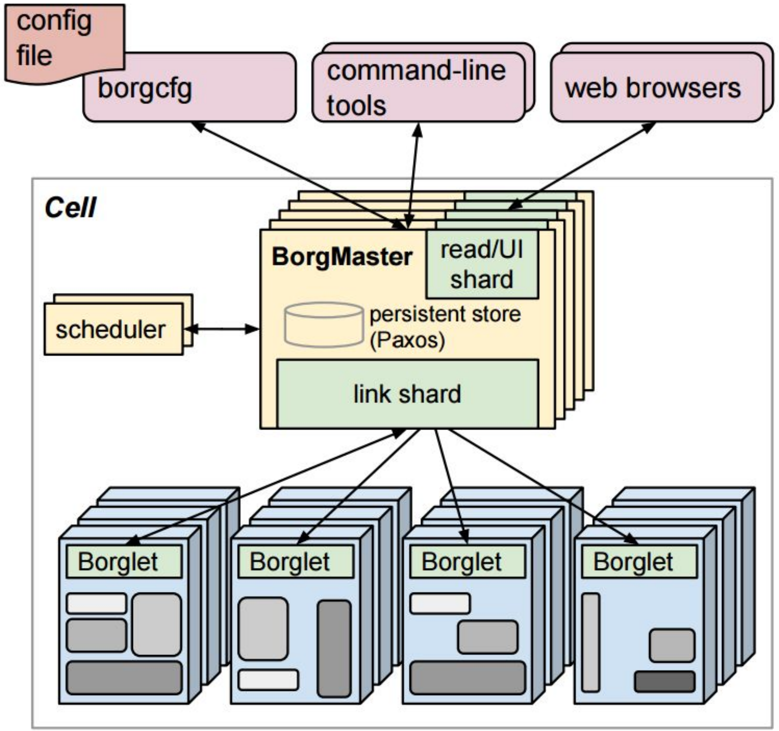

[1] http://research.google.com/pubs/pub43438.html

Growing with YouTube

- YouTube stats[1]
  - >1B users
  - 400 hrs/min of new videos (24K s/s)
  - 80% of views from outside U.S.
- Schema constantly evolving
  - Content ID, channel admin, live streams, video editing, music
- Developed live resharding

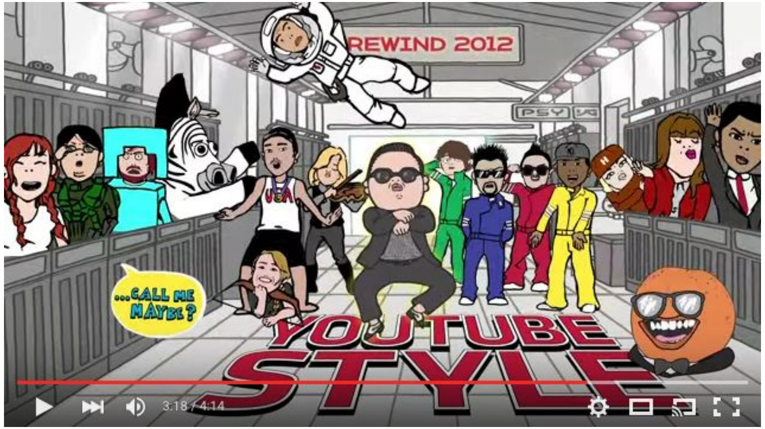

[1] youtube.com/yt/press/statistics.html

Vitess 2.0

Vitess in 2014

- Worked great in YouTube, but outside…
  - Users can't get past build step
  - Custom patched MySQL
  - No docs for setting up a deployment
  - Only client is Python
- Along comes Kubernetes
  - Like open-source Borg

Vitess in 2015

- BYO MySQL 5.6 or MariaDB 10
- Docker images
- Out-of-the-box deployment config
  - Anywhere Kubernetes runs (AWS, GCP, rackspace, …)
- Step-by-step guides
- Clients in Python, PHP, Java, Go

Vitess in 2016...

- Moving toward drop-in MySQL compatibility (Vindexes, drivers)

Vitess Terminology

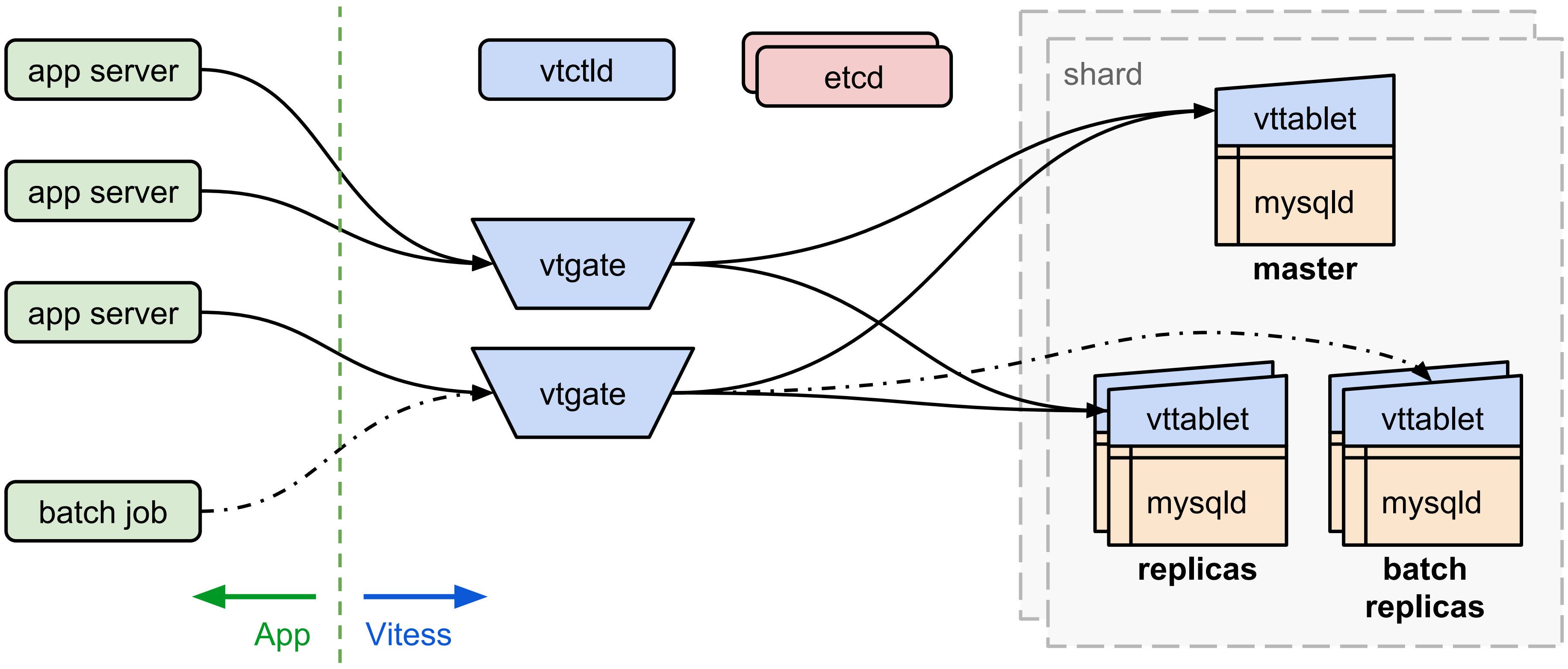

Kubernetes

Kubernetes (k8s)

kubernetes.io

- Provides glue for distributed apps
    - Dynamic scheduling
    - Declarative deployment config
    - Service discovery
    - High-availability replica pools
    - Rolling updates
    - Component grouping/metadata
- Abstracts cloud-platform-specific pieces
    - VM instance creation and config
    - Networking config
    - External load balancers
    - Persistent storage volumes (PD, EBS)

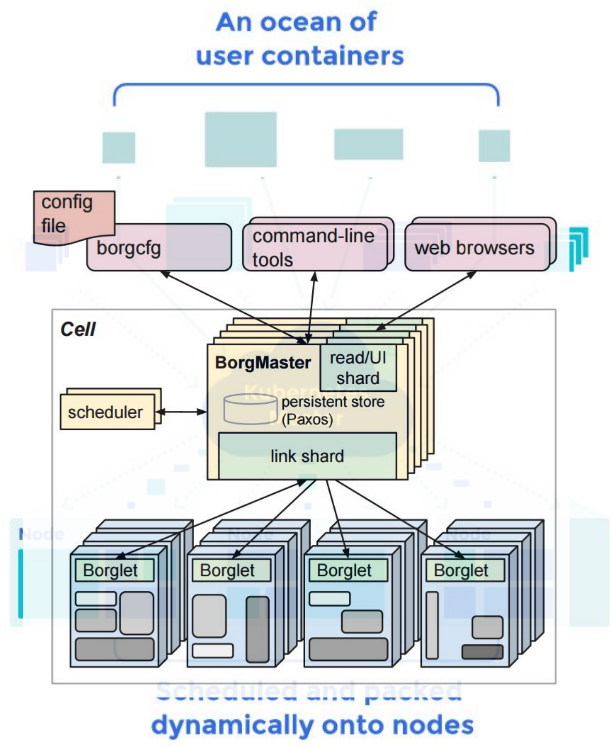

Kubernetes Terminology

- **Node** - Physical machine or VM, member of k8s cluster
- **Container** - Docker, rkt
- **Pod** - Scheduling unit (group of containers)
- **Volume** - Storage mounted into pods/containers
- **Replication Controller** - Ensures a pool of N copies of a pod template.
- **Service** - Discovery + LB Proxy based on Labels.

Databases on Kubernetes

Pets vs. Cattle[1]

Your servers might be **pets** if...	Your servers might be **cattle** if...
You can log into them by hostname or IP.	You know only that a bunch of them are out there, somewhere.
You load data onto them and tell them what to do.	They go off and join the herd without being told to.
You try to fix them when they go down.	You wait for others to take over their jobs when they go down.
Your app knows which servers to send which queries to.	Your app throws queries over a magic wall and results appear.
You know when your last master failover was.	You have a monitoring graph of failovers per day.

[1] Noah Slater, blog.engineyard.com/2014/pets-vs-cattle

Cattle Databases

- Why do we want to run databases in Kubernetes?
  - It's how we run them in Google (Borg)
  - Resource utilization
  - Horizontal scalability
  - Cluster management
- Why is it hard?
  - Make 1000s of servers look like 1 DB to the app
  - Pods are, by design, not durable
  - Durability comes only from pools of replicas

Where do you put data in Kubernetes?

- emptyDir
  - Local, ephemeral
- hostPath
  - Local, node-specific
- gcePersistentDisk, awsElasticBlockStore
  - Manually create one for every pod
- Persistent Volumes
  - PV : EBS :: node : VM
  - Pool of PVs
  - PV Claim (PVC)
  - PVC : PV :: pod : node
  - Compute and storage decoupled

Pet Set Proposal [1]

Replication Controller

- Ensure there are N **identical** copies of this pod template.

Pet Set

- Ensure there are N **similar** copies of this pod template, such that:
  - At most 1 has ID 0
  - At most 1 has ID 1
  - ...
  - (at any point in time)

[1] https://github.com/kubernetes/kubernetes/pull/18016

Vitess on Kubernetes

vitess.io/getting-started

Vitess Terminology Review

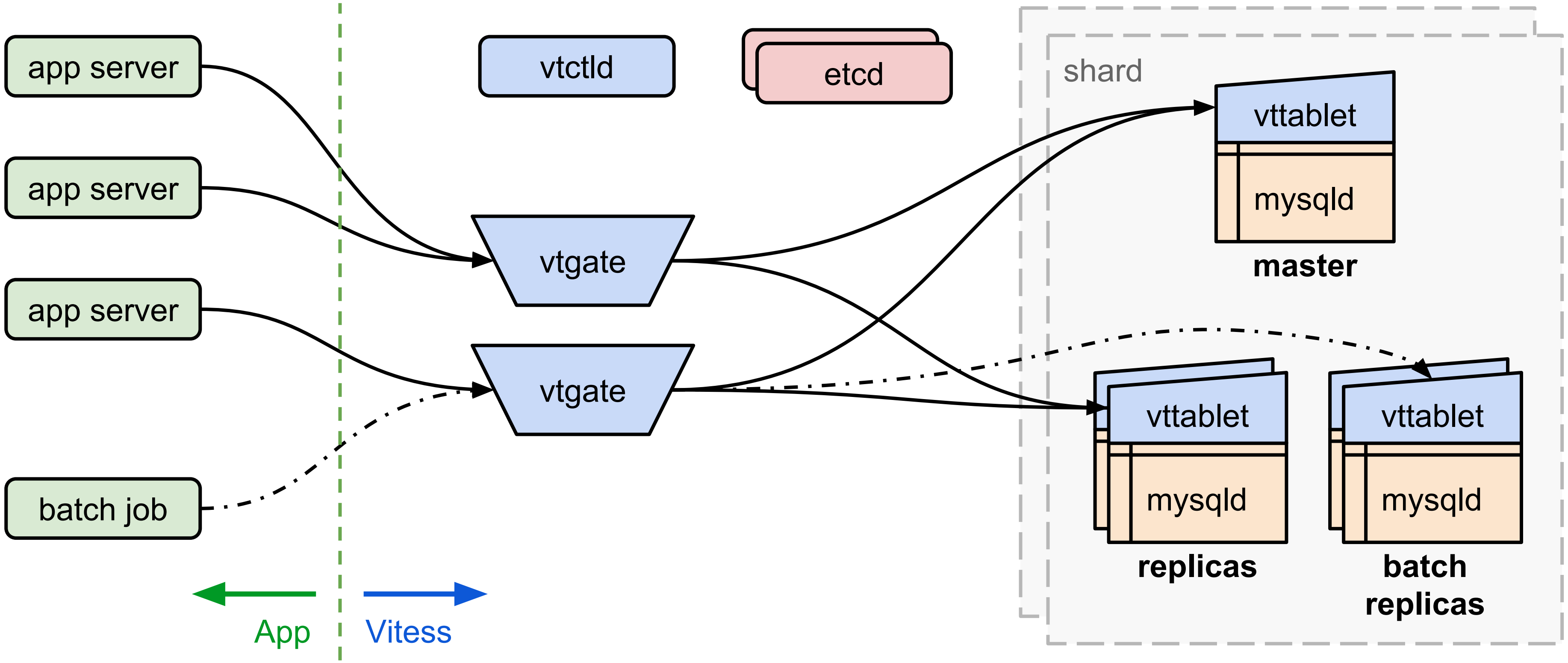

etcd (distributed config)

- Replication Controller
- Crazy bootstrap script
  - ETCD_DISCOVERY_SRV
  - Make identical replicas do different things (needs PetSet!)
- Service: etcd-global
- DNS: http://etcd-global:4001

```
ipaddr=$(hostname -i)
peer_url="http://$ipaddr:7001"
client_url="http://$ipaddr:4001"

export ETCD_NAME=$HOSTNAME
export ETCD_DATA_DIR=/vt/vtdataroot/etcd-$ETCD_NAME
export ETCD_STRICT_RECONFIG_CHECK=true
export ETCD_ADVERTISE_CLIENT_URLS=$client_url
export ETCD_INITIAL_ADVERTISE_PEER_URLS=$peer_url
export ETCD_LISTEN_CLIENT_URLS=$client_url
export ETCD_LISTEN_PEER_URLS=$peer_url

if [ -d $ETCD_DATA_DIR ]; then
  # We've been restarted with an intact datadir.
  # Just run without trying to do any bootstrapping.
  echo "Resuming with existing data dir: $ETCD_DATA_DIR"
else
  # This is the first run for this member.

  # If there's already a functioning cluster, join it.
  echo "Checking for existing cluster by trying to join..."
  if result=$(etcdctl -C http://etcd-{{cell}}:4001 member add $ETCD_NAME $peer_url); then
    [[ "$result" =~ ETCD_INITIAL_CLUSTER=\"([^\"]*)\" ]] && \
    export ETCD_INITIAL_CLUSTER="${BASH_REMATCH[1]}"
    export ETCD_INITIAL_CLUSTER_STATE=existing
    echo "Joining existing cluster: $ETCD_INITIAL_CLUSTER"
  else
    # Join failed. Assume we're trying to bootstrap.

    # First register with global topo, if we aren't global.
    if [ "{{cell}}" != "global" ]; then
      echo "Registering cell "{{cell}}" with global etcd..."
      until etcdctl -C "http://etcd-global:4001" \
          set "/vt/cells/{{cell}}" "http://etcd-{{cell}}:4001"; do
        echo "[$(date)] waiting for global etcd to register cell '{{cell}}'"
        sleep 1
      done
    fi

    # Use DNS to bootstrap.

    # First wait for the desired number of replicas to show up.
    echo "Waiting for {{replicas}} replicas in SRV record for etcd-{{cell}}-srv..."
    until [ $(getsrv etcd-server tcp etcd-{{cell}}-srv | wc -l) -eq {{replicas}} ]; do
      echo "[$(date)] waiting for {{replicas}} entries in SRV record for etcd-{{cell}}-srv"
      sleep 1
    done

    export ETCD_DISCOVERY_SRV=etcd-{{cell}}-srv
    echo "Bootstrapping with DNS discovery:"
    getsrv etcd-server tcp etcd-{{cell}}-srv
  fi
fi

# We've set up the env as we want it. Now run.
etcd
```

github.com/youtube/vitess/tree/master**/examples/kubernetes**

vtctld (admin interface)

- Replication Controller
- Service
- kubectl proxy
  - Web UI
- kubectl port-forward
  - CLI (gRPC.io)

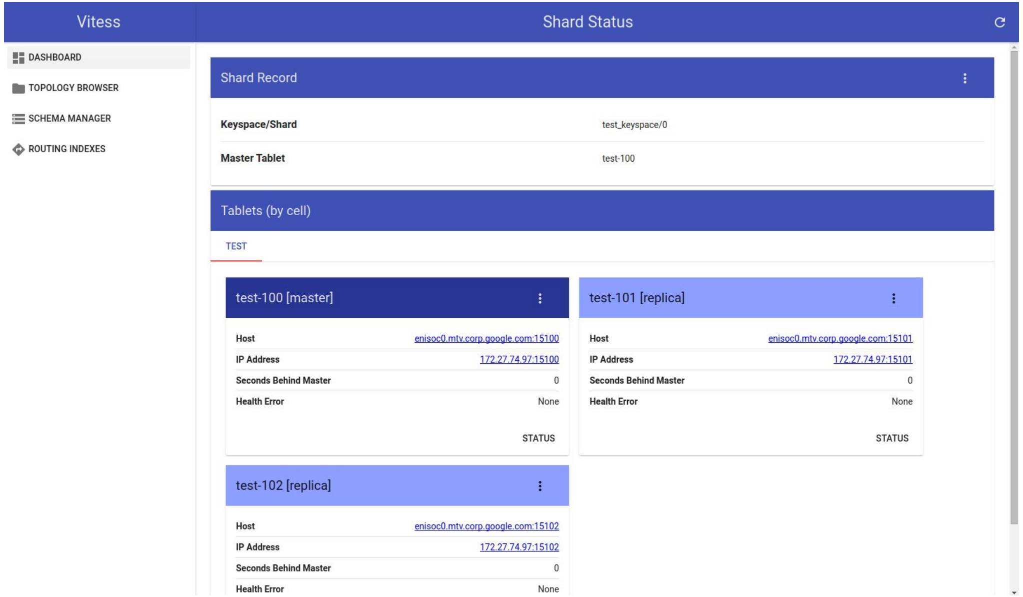

vtgate (client entrypoint)

- Replication Controller
- Service
- DNS

```
conn = vtgatev2.connect({'vt': ['vtgate:15001']}, timeout)
```

vttablet (the hard part)

- Pod
  - vttablet container
  - mysqld container
- No RC yet...
  - Containers restart
  - But pods don't get rescheduled
- emptyDir
  - Shared by both containers
  - It's... an empty dir.
  - Where does the data come from?
- Vitess Backup/Restore
  - Pulls latest snapshot from GCS/NFS

Sneak Peeks

Toward Drop-in Sharding (Vindexes)

- vitess.io/doc/VTGateV3Features
- VTGate parses and understands queries.

```
def my_hash(user_id):
  m = hashlib.md5()
  m.update(uint64.pack(user_id))
  return m.digest()[:8]
```

```
cursor = conn.cursor('test_keyspace', 'replica', keyspace_ids=[my_hash(user_id)])
cursor.execute(
  'SELECT message FROM messages WHERE user_id=%(user_id)s ORDER BY time_created_ns',
  {'user_id': user_id})
return [row[0] for row in cursor.fetchall()]
```

Automated Master Election (Orchestrator)

- Orchestrator
  - by Shlomi Noach (GitHub, formerly Booking.com)
  - github.com/outbrain/orchestrator

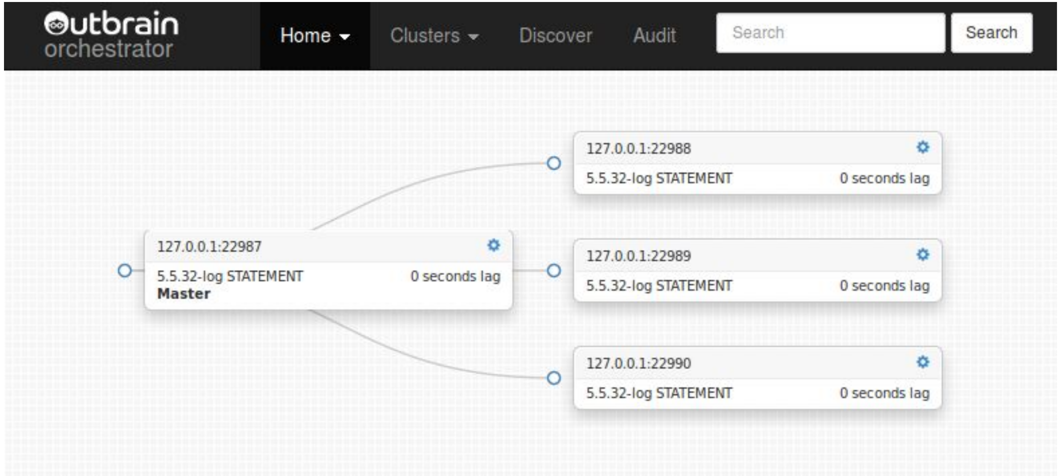

Resources

Try Vitess

vitess.io/getting-started

vitess.io/user-guide/sharding-kubernetes.html

Contribute

github.com/youtube/vitess

Contact Us

vitess@googlegroups.com

Get Updates

groups.google.com/d/forum/vitess-announce

Cloud Native Computing Foundation

cncf.io

Kubernetes

kubernetes.io
